# Participation in the convention on migratory species: A biogeographic assessment

**DOI:** 10.1007/s13280-018-1024-0

**Published:** 2018-02-24

**Authors:** Christopher Michael Hensz, Jorge Soberón

**Affiliations:** Department of Ecology and Evolutionary Biology and Biodiversity Institute, University of Kansas, 1345 Jayhawk Blvd, Lawrence, KS 66045 USA

**Keywords:** Conservation policy, Convention on Migratory Species, Multilateral Environmental Agreement, Spatial ecology

## Abstract

The Convention on the Conservation of Migratory Species of Wild Animals (CMS) is a Multilateral Environmental Agreement (MEA) focused on species that regularly travel across international borders. Despite covering an important group of species, CMS is under-utilized compared to other conservation-focused MEAs. CMS suffers from a lack of participation across North America and most of Asia. Our goal is to illustrate differences in species richness and average range-size across signatory and nonsignatory nation-states using range–diversity plots. We also show differences in the cost of CMS membership relative to species patterns to highlight which countries may be discouraged from becoming CMS signatories. Despite containing many CMS species, large economies such as the United States, Russia, and China are not members of the convention. To facilitate migratory species conservation into the future, CMS should seek to fill gaps in participation, potentially directing recruitment efforts toward nonsignatory states that would receive the largest benefit at the lowest relative cost.

## Introduction

Multilateral Environmental Agreements (MEAs) are legally binding instruments between two or more nation-states that address environmental issues (Dodds et al. 2007). Approximately, 700 international agreements can be identified as MEAs (Kim 2013). According to Koester (2002), the most important MEAs concerning biodiversity conservation are the Convention on Biological Diversity (CBD 1992), the Convention on International Trade in Endangered Species of Fauna and Flora (CITES 1973), the Convention on Wetlands of International Importance (RAMSAR 1971), the Convention Concerning the Protection of the World Cultural and Natural Heritage (WHC 1972), and the Convention on the Conservation of Migratory Species of Wild Animals (CMS 1979). Of these, the CBD is regarded as the most politically salient, and CITES the most operative in administrative regulation (Guruswamy 1999). Despite the ecological importance of transboundary species movements (Clobert et al. 2012), CMS is the only MEA focused broadly on migratory species across taxonomic divisions. Unlike CITES, CMS lacks stringent participation requirements for party states. Instead, CMS operates by facilitating the creation of smaller cooperative agreements (Seelarbokus 2014), including as many as 106 “action plans” across seven major conservation agreements and 19 nonbinding Memoranda of Understanding (MoUs). These agreements under CMS administration have helped to stabilize populations of migratory species including Wadden Sea seals (*Phoca vitulina vitulina and Helichoerus grypus*) and the Bukhara deer (*Cervus elaphus bactrianus*) despite being nonbinding (Baldwin 2011). Since its initial signing in 1979, CMS membership increased from 29 signatories to 126 party states by 2017 (Guruswamy and Doran 2007; Birnie et al. 2009).

CMS defines migratory species as those “whose members cyclically and predictably cross one or more national jurisdictional boundaries” (CMS 1979). CMS also covers several species that cross international borders but are nonmigratory such as marine otters (*Lontra felina*) and mountain gorillas (*Gorilla gorilla*). CMS lists migratory species in two appendices as agreed upon by party states: Appendix I includes endangered species restricted from taking (harvesting, hunting, etc.), Appendix II lists species with unfavorable conservation status that may benefit from international cooperation, but are not restricted from taking. Several levels of biological organization are listed in each appendix (genera, species, subspecies, and populations) and these groups may be included in either or both appendices (CMS Appendix I and II, updated October 2017).

Becoming a party to CMS represents a large investment of expertise and time. CMS signatories agree to (i) undertake active conservation of migratory species under the first appendix of the agreement, (ii) form additional international agreements to conserve species in the second appendix, (iii) participate in the tri-annual Conference of the Parties, and (iv) financially support the CMS secretariat (CMS 1979). A significant obstacle to encouraging large, economically powerful states to joining the convention is the cost of being a signatory. Similar to the General Assembly of the United Nations, the cost of participation in CMS is weighted by the GDP of signatory states (UNEP/CMS Res 12.2).

In this contribution, we aim to describe CMS from a biogeographic perspective to identify which countries may be most amenable to becoming signatories. We analyze the geographic structure of the species covered under CMS Appendix I and II using range–diversity plots (Arita et al. 2008) and relate the results of these plots to United Nations (UN) economic indices as a measure of participation cost. We aim to provide international policymakers the tools to evaluate the potential conservation benefits of joining CMS.

## Materials and methods

We obtained the full record of 1115 CMS species through Species+, a database of CMS and CITES species (http://speciesplus.net; accessed March 2nd 2017). We aggregated the data to include a single record for each species, consolidating all species with multiple listed subpopulations and species under both CMS appendices. Sixty-two species included no range data and were excluded from this analysis. Species+ database lists the countries where each species is found, but has no data for geographic range size by country, limiting analysis to the country scale.

The Holy See and South Sudan were excluded from analysis for poor data quality: The Holy See contains zero records, and South Sudan could not be completely distinguished from Sudan in the database. Consequently, the maps we present depict a single united Sudan, reflecting the resolution of species data rather than political reality. Greenland was excluded from analysis as it has no established relationship with CMS and is independent of Denmark in its conservation decisions. The Cook Islands and Niue, despite being technically in association with New Zealand, have signed CMS independently and are thus treated as independent for this study (http://www.cms.int/en/parties-range-states; accessed March 2, 2017). For all other countries, we aggregated species data to the level of sovereign states, including all territories under each country (including American Samoa for the United States, French Guiana for France, etc.).

We used two sources of data to determine the economic cost of being a party to CMS. For most signatory states, the financial contribution of each country from 2018–2020 is presented in reports from the 12th Conference of Parties in 2017 (COP12; UNEP/CMS/Resolution 12.2, pp. 5–8). Parties to CMS contribute funding proportional to the size of their respective economies, measured in gross domestic product (GDP). To estimate the cost for a non-party to become a member of CMS, we added proportional 2018 GDP estimates for individual non-party states obtained from the United Nations General Assembly (UNGA Res A/70/416/Add.1, pp. 3–8) to the CMS document and calculated cost based on the new proportional GDP. We obtained the signatory status of each country and designation of sovereign territories through the CMS web page (http://www.cms.int/en/parties-range-states; accessed March 2nd 2017).

We characterized species patterns for each country using richness–diversity diagrams, a biogeographic exploratory tool (Arita et al. 2008; Soberón and Ceballos 2011) grouping the plots by (i) *k*-means clustering (MacQueen 1967) and (ii) CMS geographic region. *k*-means clustering of the range–diversity plots divides countries into groups that have similar properties based upon species-level patterns. Alternatively, grouping by the six CMS geographic regions (North America, Europe, Asia, Australia and Oceania, Africa, and South America, and the Caribbean) indicates whether or not geographic proximity plays a dominant role in CMS species patterns. Richness–diversity diagrams use the presence–absence data to describe species compositions of each recorded location in a dataset. From these diagrams, it is possible to extract biodiversity indices including alpha and beta diversities (Soberón and Cavner 2015). The horizontal axis shows the proportional mean range size, also called the dispersal field, of the species in each location (Graves and Rahbek 2005). Proportional mean range size (referred from here on as simply ‘range-size’), indicates how cosmopolitan species are for each location. For example, if a country has a relatively large range-size value (e.g., > 0.75), species within that country occur in at least 75% of countries on average; further, a range-size value of 1 means that all species in that country are represented globally. The minimum possible range-size value, 1/*n*, (where *n* is the number of sites) indicates that all species present in a country are endemic and thus nonmigratory. Calculations were performed in R (R Core Team 2017), and the resulting maps were created in ArcGIS (ESRI 2011).

## Results and discussion

**Fig. 1:**
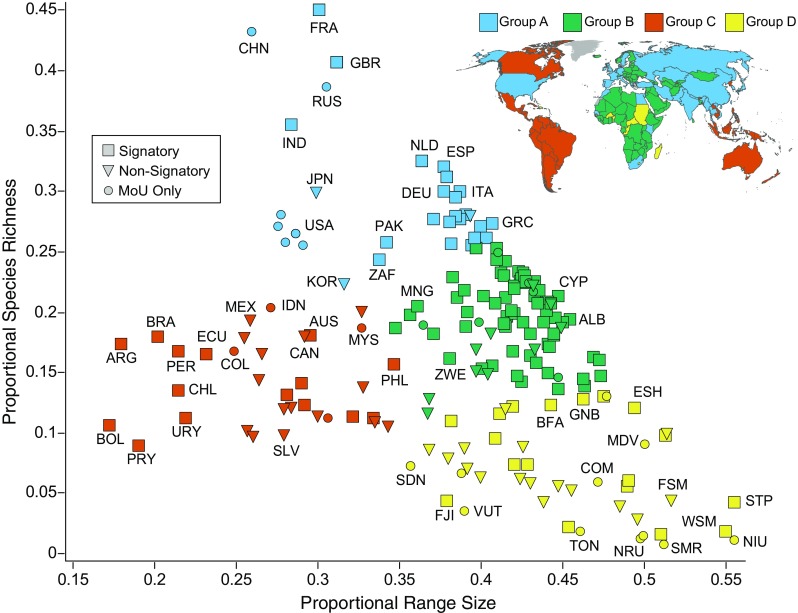
Richness–diversity diagram and map of countries grouped by *k*-means clustering. Diagram depicts the relationship between the number of CMS species in a country and the average range-size of those species. Group A is in light-blue, group B is in green, group C is in red, and group D is in yellow. Each point represents a sovereign country and all of its territories. Signatory states are indicated by squares, nonsignatory states are indicated by triangles, and countries that have signed at least one MoU but not CMS are indicated by circles. Select countries are labeled on the plot with three-letter country codes

K-means clustering analyses identified four distinct groups of countries (referred to as groups A, B, C, and D; Fig. 1). Group A includes 32 countries with the largest number of CMS species, the five with the most species being France, China, Great Britain, Russia, and India (Table 1). Both Great Britain and France are sovereign over territories in multiple hemispheres (including sub-Antarctic island territories), inflating the overall number of species observed for those countries. India, Russia, and China also contain a large number of CMS species (> 350), perhaps due to large geographic extent. Despite participating in at least one MoU administered by the CMS secretariat and being the second and fourth largest hosts of CMS species, respectively, neither China nor Russia is currently members of CMS. Nonsignatory countries in group A may be more amenable to joining CMS signatories as they already contain many species listed under CMS.

**Table 1:** Summary statistics of species richness and mean range-size of countries by group

Grouping method	Group/region	Number of countries	Mean number of CMS species	Mean range-size of species (number of countries)	Number of signatory countries
*k*-means clustering	A	32	300.7	66.7	22 (68.7%)
	B	83	201.9	80.4	66 (79.5%)
	C	34	148.9	52.3	15 (44.1%)
	D	46	74.0	86.5	18 (39.1%)
Geographic region	North America	3	223.0	53.5	0 (0%)
	Europe	48	231.1	80.4	41 (85.4%)
	Asia	39	227.4	71.3	16 (41.0%)
	Australia and Oceania	19	66.2	82.2	7 (36.8%)
	Africa	54	177.2	80.0	44 (81.5%)
	South America and the Caribbean	32	114.8	32.0	13 (40.6%)

Group B comprises 83 countries across Europe, Africa, and Central Asia forming the center mass of the richness–diversity diagram. Group B contains the largest proportion of signatory states of any group (79.5%) and contains countries with moderate species richness and range-size values. Countries in group B on average contain fewer CMS species than countries in group A, but both groups contain species with moderate range-sizes, found in approximately 30–40% of countries worldwide (Table 1).

Group C encompasses 34 countries across North America, South America, Southeast Asia, Australia, and Oceania with CMS species that are more restricted in range-size. These species occur in relatively fewer countries (< 35%), less than 75% of all other CMS species. Many countries in group C (65.9%) are not CMS parties (notably Indonesia, Canada, and Mexico). However, because species in this group tend toward smaller range-sizes, relevant countries may be more inclined to focus on smaller, local conservation initiatives rather than on a larger multilateral agreement like CMS. From a conservation perspective, each country in group C represents a large portion of the distribution of CMS species in that region such that species in this group depend on more constrained areas. Each nonsignatory country in group C may significantly limit the effectiveness of the convention as a conservation tool for this group.

The 46 countries clustered in group D have the smallest average number of CMS species—approximately one quarter of the species found in group A (Table 1). Composed predominantly of island states alongside a few African and very small European states, each of the countries in group D contain < 15% of CMS species which are shared with 35–55% of other United Nations member countries. Countries in this group that are not already signatories may be difficult to recruit to CMS as, not only are there few CMS species in these countries, but the species in group D countries also tend to be fairly cosmopolitan, which reduces the impact of a single state’s participation. Many countries in this group are geographically restricted in size and in immediate proximity of other small states. It is also important to note that species occurring in many countries may still occur in relatively small land area depending on the geographic region in question.

**Fig. 2:**
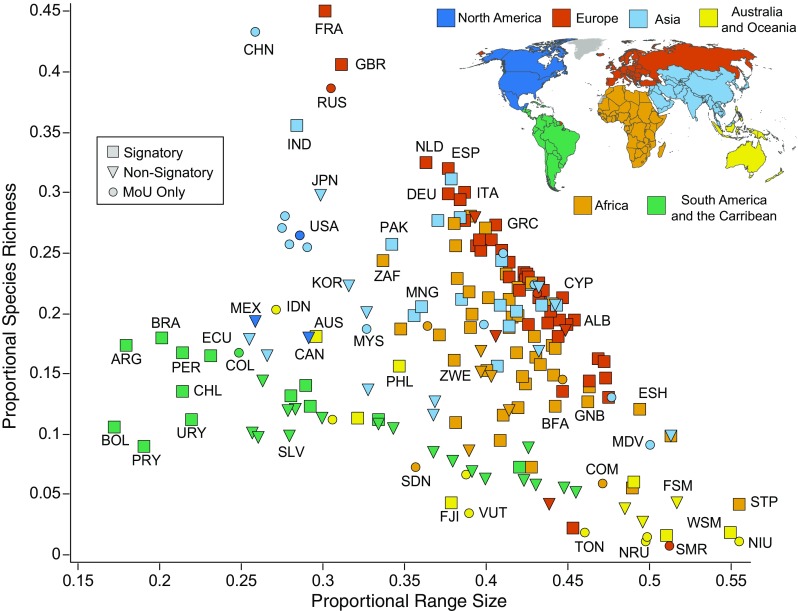
Richness–diversity diagram of countries grouped by CMS geographic regions. Diagram depicts the relationship between the number of CMS species in a country and the average range-size of those species. North America is in dark-blue, Europe is in red, Asia is in light-blue, Australia and Oceania are in yellow, Africa is in orange, South America and the Caribbean are in green. Each point represents a sovereign country and all of its territories. Signatory states are indicated by squares, nonsignatory states are indicated by triangles, and countries that have signed at least one MoU but not CMS are indicated by circles. Select countries are labeled on the plot with three-letter country codes

When looking at the range–diversity diagram with a geographic (as opposed to species-based) perspective, new patterns emerge (Fig. 2). Europe, Asia, and North America contain large numbers of CMS species, while South America and the Caribbean, and Australia and Oceania contain relatively fewer listed species (Table 1). Each geographic region forms visually identifiable clusters on the range–diversity diagram. Notable exceptions to this include Caribbean countries and very small European states (e.g., San Marino). Unsurprisingly, these countries have similar properties to small Oceanic states than large mainland states. Range–diversity diagrams grouped by geography alone may over-generalize countries that are in close proximity but have dissimilar species patterns.

**Fig. 3:**
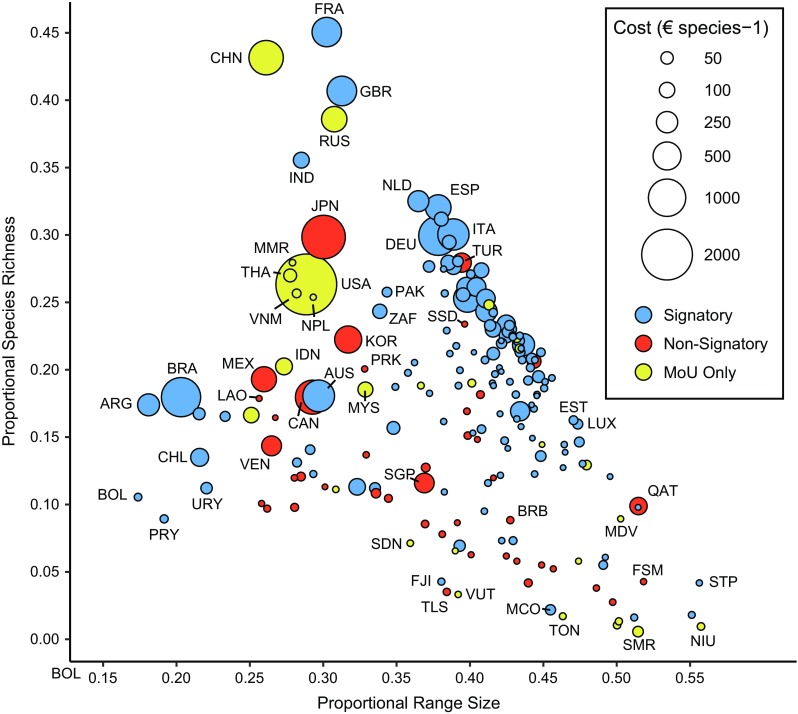
Richness–diversity diagram depicting the relationship between the number of CMS species in a country and the average range-size of those species. Each point represents a sovereign country and all of its territories. The size of each point shows the per-species cost to be a CMS party state. Signatory states are blue, nonsignatory states are indicated by red, and countries that have signed at least one MoU but not CMS are indicated by yellow. Select countries are labeled on the plot with three-letter country codes

For the 2018–2020 budgetary period, 15 states will pay the minimum contribution (less than €60 year^−1^), while the top four of the contributors (Germany, France, the United Kingdom, and Italy) will each pay more than €200 000 year^−1^ (UNEP/CMS Res 12.2, pp. 5–8; Table 2). The per-species cost to becoming a signatory is at least 14% higher for the richest nonsignatory states (the Unites States and Japan) than any current signatory state (Fig. 3, Tables 2, 3). The remaining largest nonsignatory states, China and Russia, have comparable per-species costs to the largest signatory countries. Among nonsignatory states, Myanmar, Thailand, Nepal, Vietnam, and Turkey stand out in particular (Fig. 3, Table 2) as countries containing a large proportion of CMS species (> 25%) and with relatively low participation costs (< €250 species^−1^ year^−1^).

**Table 2:** Expectation of financial contribution to CMS from nonsignatory states containing at least 25% of CMS species to become signatories based on proportional economic size (UNGA Res 70/416/Add.1, UNEP/CMS Res 12.2). Adjusted scale includes all signatory states

Country	CMS species	UN contribution scale (%)	Adjusted scale (%)	Signatory cost (€)	Cost per species (€)
China	454	7.92	14.25	364 907	803.76
Russia	406	3.09	6.10	156 192	384.71
Japan	314	9.68	16.87	431 918	1375.54
Myanmar	294	0.01	0.02	539	1.83
Turkey	294	1.02	2.10	53 745	182.81
Thailand	284	0.29	0.61	15 603	54.94
USA	277	22.00	31.42	804 453	2904.17
Vietnam	270	0.06	0.12	3126	11.58
Nepal	267	< 0.01	0.01	324	1.21

**Table 3:** Estimation of financial contribution to CMS from the 10 signatory states containing the largest number of CMS species based on proportional economic size (UNGA Res 70/416/Add.1, UNEP/CMS Res 12.2). Adjusted scale includes only signatory states

Country	CMS species	UN contribution scale (%)	Adjusted scale (%)	Signatory cost (€)	Cost per species (€)
France	474	4.86	10.24	262 177	553.12
UK	428	4.46	9.41	240 810	562.64
India	374	0.74	1.55	39 766	106.33
Netherlands	342	1.48	3.12	79 964	233.81
Spain	337	2.44	5.15	131 817	391.15
Israel	328	0.43	0.91	23 202	70.74
Italy	316	3.75	7.90	202 231	639.97
Germany	315	6.39	13.47	344 732	1094.39
Portugal	310	0.39	0.83	21 151	68.23
Egypt	295	0.15	0.32	8201	27.80

## Conclusions

While most countries in Europe, Africa, and South America are members of CMS, there are gaps in participation across Asia and North America. Countries containing a large number of CMS species, particularly those with low participation costs such as Myanmar, Thailand, Nepal, Vietnam, and Turkey may be most amenable to joining CMS. In contrast, cost may be a deterrent for nonsignatory states with large economies, particularly for those countries containing few CMS species. Regardless, CMS must not ignore the importance of pursuing geographically large nonsignatory countries that contain many species under the convention (e.g., Russia, China, Japan, and the United States). Of these countries, Russia and China would contribute comparable per-species cost to current signatory states with similar species compositions (e.g., France and the United Kingdom). The United States and Japan may be discouraged by disproportionately large costs necessary to become signatories. This cost burden may be alleviated with the addition of migratory species into CMS appendices with ranges in these countries.

For this study, the identity of individual species was not considered. However, it should not be assumed that all CMS species present equivalent conservation problems. CMS includes mammals, birds, retiles, fish, and one insect with diverse ecologies, modes of movement, and migratory habits in both terrestrial and aquatic environments. Species counts are useful for broad summaries, but it is unlikely that all species are valued equally by range-states.

The only insect listed under CMS, the monarch butterfly, is a prime example of the difficulties the convention faces with conservation of migratory species across nonsignatory states. Monarch butterflies exhibit a wide geographic range including North America, Central and South Americas, Oceania, and Australia, Europe, and Africa, but only North American populations of monarch butterflies are migratory (Zhan et al. 2014). Canada, the United States, and Mexico are not parties to CMS, preferring instead to maintain independent initiatives (Oberhauser et al. 2008). While it is possible for CMS to facilitate conservation efforts of the monarch butterfly as a species, the convention has limited ability to conserve populations of monarch exhibiting migratory behavior with no North American signatory states.

Limitations in species distribution data restrict the efficacy of any conservation assessment (Seelarbokus 2014). Distributions of migratory species are particularly difficult to catalog given their complicated and seasonal life histories (Riede 2004). The coarseness of available range data limited this study to a country-scale evaluation addressing only species included within CMS appendices. Future assessments of species composition patterns would benefit greatly from measures of geographic range and seasonality of movements.

The primary goal of the CMS secretariat is to facilitate cooperation and communication between member states in conservation efforts of migratory species that travel across international borders. CMS does not place stringent legal requirements upon its signatories unlike other MEAs such as CITES or CBD. Rather, CMS encourages the creation of smaller agreements that may themselves contain strict requirements. This approach appeals to states that opposed broad restrictions, but may hinder the efficacy of implementing localized conservation plans and protections (Baldwin 2011). CMS must focus on filling geographic gaps in participation for the agreement to be relevant on the international scale. Large geographic gaps in participation discourage nonsignatory states in North America and Asia from entering CMS on an individual basis. Nonsignatory countries may contain ecological regions critical to the conservation of a migratory species such as breeding sites, migratory flyways, stopovers, or wintering areas. Moreover, as global climate change influences migration patterns (Robinson et al. 2009), CMS may become increasingly important as an MEA. Without adequate participation from the global community, CMS is ultimately limited in its ability to facilitate conservation of migratory species.
